# Gold Nanoparticles/Nanographene-Based 3D Sensors Integrated in Mini-Platforms for Thiamine Detection

**DOI:** 10.3390/s23010344

**Published:** 2022-12-29

**Authors:** Damaris-Cristina Gheorghe, Jacobus (Koos) Frederick van Staden, Raluca-Ioana Stefan-van Staden, Paula Sfirloaga

**Affiliations:** 1 Laboratory of Electrochemistry and PATLAB, National Institute of Research for Electrochemistry and Condensed Matter, 202 Splaiul Independentei Str., 060021 Bucharest, Romania; 2 Faculty of Chemical Engineering and Biotechnologies, University Politehnica of Bucharest, 060021 Bucharest, Romania; 3 National Institute for Research and Development in Electrochemistry and Condensed Matter, Dr. Aurel Paunescu Podeanu 144, 300569 Timisoara, Romania

**Keywords:** thiamine, electrochemical sensors, graphene, food and water analysis, biomedical analysis

## Abstract

Vitamins are essential for sustaining daily activities and perform crucial roles in metabolism, such as preventing vascular events and delaying the development of diabetic nephropathy. The ultrasensitive assessment of thiamine in foods is required for food quality evaluation. A mini-platform utilizing two 3D sensors based on nanographene and gold nanoparticles paste modified with protoporphyrin IX and protoporphyrin IX cobalt chloride is proposed for the detection of thiamine in blueberry syrup, multivitamin tablets, water, and a biological sample (urine). Differential pulse voltammetry was utilized for the characterization and validation of the suggested sensors. The sensor modified with protoporphyrin IX has a detection limit of 3.0 × 10^−13^ mol L^−1^ and a quantification limit of 1.0 × 10^−12^ mol L^−1^, whereas the sensor modified with protoporphyrin IX cobalt chloride has detection and quantification limits of 3.0 × 10^−12^ and 1.0 × 10^−11^ mol L^−1^, respectively. High recoveries (values greater than 95.00%) and low RSD (%) values (less than 5.00%) are recorded for both 3D sensors when used for the determination of thiamine in blueberry syrup, multivitamin tablets, water, and urine, demonstrating the 3D sensors’ and suggested method’s high reliability.

## 1. Introduction

Thiamine, also known as vitamin B1, is part of the vitamin B complex and plays a role in the development of the brain and neurons [1]. The human body stores at least 30 g worth of vitamin B1 at any given time [2]. Although it is a water-soluble vitamin, it cannot be stored in the human body because it travels with water and is excreted from the body through urine; therefore, it must be ingested regularly [3]. The deficiency of thiamine can lead to beriberi, which can have detrimental effects on the neuron system. Consuming foods rich in thiamine from any source or supplement may serve as a means of protection against the condition. As vitamin B1 plays such an important role in the human body, researchers developed methods to determine whether or not foods or particular supplements contain it [4]. Methods such as high-performance liquid chromatography [1] and fluorescence [5] are just a few of the methods that have been developed. These techniques are superior in that they have a high accuracy and coefficient of determination, in addition to a low detection limit. Nevertheless, these techniques come with a few drawbacks, such as time-consuming sample preparation, the need for expensive high-tech equipment, and a high price tag. These issues could be circumvented by employing an electrochemical technique, specifically voltammetry. These techniques have a number of benefits, some of which are listed below: sensitivity, the ability to generate data that can be interpreted even at low concentration levels, speed, ease of use, and a low-cost analysis [6]. Electrochemical detection of thiamine is possible due to the oxidizable nature of the molecule [4,6].

Electrochemical techniques are extremely useful in the development of sensors for health-related applications [7]. Electrochemical sensors measure the current of the working electrode, which is based on the electrochemical redox (oxidation/reduction) process of an analyte. This allows the sensors to record the concentration of an analyte of interest that is present in the electrolyte solution, which in our case is thiamine. The magnitude of the current response is typically proportional to the concentration of the analyte. Amperometry and voltammetry are the two methods of electrochemistry that are employed most frequently today. The two voltammetry processes that are the least complicated are called cyclic voltammetry (CV) and linear scan voltammetry. Square wave voltammetry (SWV) and differential pulse voltammetry (DPV) are two methods that, with the appropriate adjustments to a variety of parameters, are capable of producing results with increased sensitivity [8].

Compared to their bulk counterparts, noble metal nanoparticles (NPs) (e.g., Ag, Au) exhibit distinct chemical and physical properties, namely, electronic, optical, magnetic, and catalytic capabilities [9]. Due to their capacity to facilitate electron transfer processes on the surface of materials used for electrocatalysis [10], and nanostructured substrates for surface-enhanced Raman scattering (SERS) [11], metal nanoparticles (MNPs) have been widely exploited in the development of electrochemical sensors. The wide applicability of these materials is a result of their biocompatibility with a variety of biomolecules [12], and their ability to provide a suitable microenvironment for the immobilization of biomolecules [13]. Au NPs have strong absorption properties, good stability, and good conductivity [14], which endows them with biosensing capability for enzyme detection [15], and immunosensors [16]; therefore, we selected them for the design of the 3D sensors. Graphene–metal nanostructure composites exhibit enhanced performance compared to NPs and graphene was considered separately due to synergistic effects. Graphene possesses exceptional physical and chemical characteristics and can, therefore, be utilized effectively in various biosensing applications [15], including the applications as matrix for the proposed 3D sensors. Similarly, nanoparticles are extensively researched in the realm of biosensing due to their unique physical and chemical properties, including quantum size and surface effect, etc. Combining NPs and graphene to generate graphene–NPs hybrid affords the hybrid materials significantly enhanced sensing characteristics, enhances the surface area for analyte binding, and boosts electron mobility and conductivity. For chemical reactions, particularly those involving electron transfer, porphyrins [10] are well-known for their electrocatalytic properties [17].

The present study illustrates the design and validation of a simple method for the quantification of thiamine in beverages, pharmaceuticals, water, and biological samples (urine). The novelty of the work is the utilization of a mini-platform that employs two 3D electrochemical sensors based on gold nanoparticles and nanographene (nGr) and modified with two types of porphyrins: protoporphyrin IX (PIX) and protoporphyrin IX cobalt chloride (PIXCoCl). These types of sensors are required due to the fact that food safety is a crucial factor in addressing dangers to human health and population well-being [18,19].

## 2. Materials and Methods

### 2.1. Chemicals

Thiamine, AuNPs, nGr, PIX, PIXCoCl, monosodium phosphate, disodium phosphate, vitamin B12, ascorbic acid, maltodextrin, fructose, glucose, ammonium chloride, iron (II) sulphate hydrate, and sodium acetate were obtained from Sigma Aldrich (Milwaukee, WI, USA). Fluka supplied the paraffin oil (d420, 0.86 g cm^−1^) (Buchs, Sweden). Phosphate buffer solution (PBS, 0.1 mol L^−1^) was made by combining monosodium phosphate and disodium phosphate in a solution-making process. To achieve the desired range of pH values (2.0, 3.0, 4.0, 5.0, 6.0, 7.0, and 8.0), the pH of the buffer solution was modified by adding varying amounts of solutions with a concentration of 0.1 mol L^−1^ of either NaOH or HCl.

For the preparation of thiamine solutions, PBS (pH 2.0) was utilized. The stock solution (10^−2^ mol L^−1^) was prepared only with PBS (pH 2.0) and had a concentration of 10^−2^ mol L^−1^. The remaining solutions (10^−3^ mol L^−1^ −10^−12^ mol L^−1^) were prepared using PBS and 0.1 mol L^−1^ NaNO_3_ as supporting electrolytes. When not in use, all solutions were stored in a dark, dry place, at room temperature.

### 2.2. Methods and Equipment

A mini potentiostat, widely recognized as the EmStat Pico, was connected to a laptop running PSTrace software version 5.9 (PalmSens BV, Houten, The Netherlands) for the purpose of acquiring data in order to carry out electrochemical measurements such as CV, DPV, and electrochemical impedance spectroscopy (EIS). A conventional three-electrode system was implemented by using PIX/AuNPsnGr and PIXCoCl/AuNPsnGr as working electrodes, an Ag/AgCl wire (1 mol L^−1^ KCl) as a reference electrode, and a Pt wire as a counter electrode. A Mettler Toledo pH meter was employed in order to make the necessary pH adjustments.

Every measurement was performed with the instrument at ambient temperature.

A qualitative analysis of the materials that were studied was carried out with the assistance of scanning electron microscopy (SEM) (Inspect S), manufactured by FEI Company Netherlands. All of the samples were analyzed using the ETD detector in high-vacuum mode with a high voltage (HV) of 25 kV and a spot value of 2 at 1600 times the magnification. This was performed so that the picture resolution could be obtained.

### 2.3. Development of the PIX/AuNPsnGr and PIX/AuNPsnGr 3D Sensors’ Designs

**Scheme 1:**
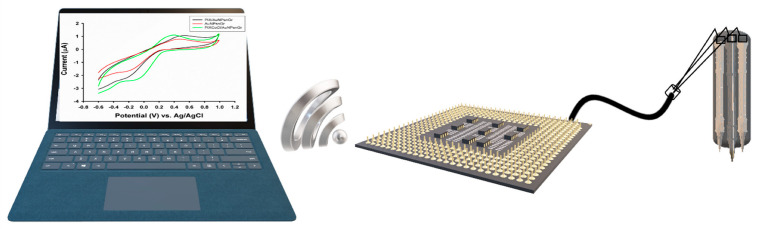
Schematic representation of the mini-platform.

In order to put together the mini-platform containing the PIX/AuNPsnGr and PIX/AuNPsnGr electrochemical sensors (Scheme 1), 100 mg of nanographene powder, 10 µL of AuNPs, a suitable quantity of paraffin oil, and 100 µL of PIX and PIXCoCl were physically mixed together in order to obtain homogenous pastes. Following the placement of the pastes into the plastic tubes, a silver wire was used to establish the electrical contact. Before performing each analysis, the electrodes were rinsed with deionized water to remove any residue. Polishing on aluminum foil until a smooth surface was attained allowed the PIX/AuNPsnGr and PIX/AuNPsnGr surfaces to be refreshed. Whenever they were not in use, the 3D sensors were kept at a temperature between 2 and 8 °C.

### 2.4. Procedure: Differential Pulse Voltammetry

Each DPV measurement was carried out within a potential domain that varied from −1.00 V to 1.00 V, a step potential that was 25.0 mV s^−1^, and a modulation amplitude that was 100 mV. The calibration curve was established by graphing thiamine concentrations ranging from 10^−3^ mol L^−1^ to 10^−10^ mol L^−1^ against their respective peak heights with a correlation coefficient higher than 0.991. The DPV peaks were given a baseline adjustment.

### 2.5. Samples

The created electrochemical sensors were utilized to determine the thiamine concentration in blueberry syrup, multivitamin tablets, water, and a healthy subject’s biological sample (urine). After diluting the samples in PBS with a pH of 2.0 using a volume-to-volume ratio of 1:1, a range of thiamine concentrations were added to the samples.

The following were performed in order to prepare the samples: after 1 g of blueberry syrup was measured using an analytical balance, the sample was diluted with 8 mL of PBS with a pH of 2.0 and 1 mL of 0.1 mol L^−1^ of NaNO_3_. Before analyzing the water, urine, and multivitamin tablet samples, 1 mL of each sample was diluted with 8 mL of PBS with a pH of 2.0 and 1 mL of 0.1 mol L^−1^ of NaNO_3_.

## 3. Results and Discussion

### 3.1. Morphological Characterization of the Pastes

**Figure 1:**
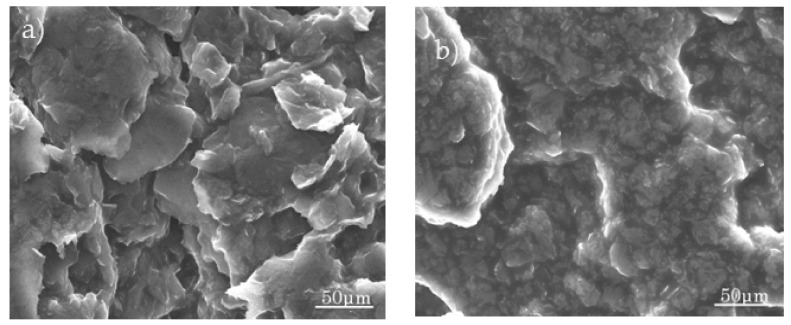

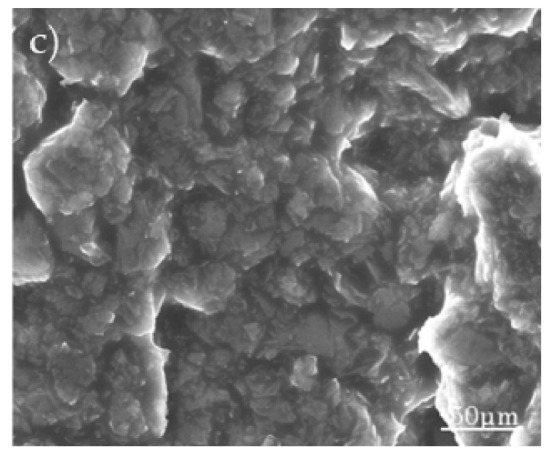
Representative SEM micrographs of: AuNPsnGr (**a**), PIX/AuNPsnGr (**b**), and PIXCoCl/AuNPsnGr (**c**).

For the purpose of determining the morphology of the material, SEM microscopy was performed. Figure 1 illustrates the surface morphology of AuNPSnGr (Figure 1a), PIX/AuNPsnGr (Figure 1b), and PIXCoCl/AuNPsnGr (Figure 1c) pastes. Figure 1a shows the surface morphology of the paste, which consists of numerous layers of irregularly shaped flakes of varying sizes. It is possible to see that the particles in the pastes that are modified with PIX and PIXCoCl, shown in Figure 1b,c, have an irregular shape and are agglomerated in asymmetric formations.

### 3.2. Electrochemical Characterization of the 3D Sensors

**Figure 2:**
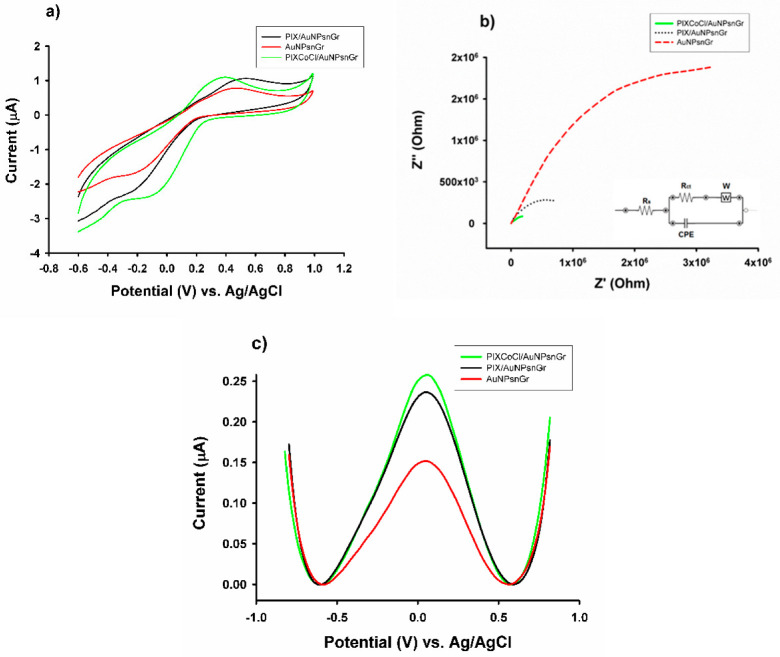
(**a**) CVs of the current versus the potential (working conditions: step potential 0.025 V; scan rate 0.1 V s^−1^) in a 5.0 × 10^−3^ mol L^−1^ K_3_[Fe(CN)_6_] (0.1 mol L^−1^ KCl) solution employing AuNPsnGr (red line), PIX/AuNPsnGr (dark line), and PIXCoCl/AuNPsnGr (green line); (**b**) EISs recorded for AuNPsnGr (red), PIX/AuNPsnGr (dark), and PIXCoCl/AuNPsnGr (green) in a 5.0 × 10^−3^ mol L^−1^ K_3_[Fe(CN)_6_] (0.1 mol L^−1^ KCl) solution (working conditions: frequency range between 10^5^ to 10^−1^ Hz). Inset: the equivalent circuit diagram of the electrochemical interface used to fit the EIS where R_s_ is the solution resistance; CPE is the constant phase element; W is the Warburg diffusion resistance; R_ct_ is the electron-transfer resistance; (**c**) DPVs recorded in pH 2.0 PBS containing 0.1 mol L^−1^ NaNO_3_ as supporting electrolyte and 1^−9^ mol L^−1^ thiamine for AuNPsnGr (red), PIX/AuNPsnGr (dark), and PIXCoCl/AuNPsnGr (green).

Electrochemical characterization was performed using two types of methods (CV and EIS) on bare (AuNPsnGr) and modified (PIX/AuNPsnGr and PIX/CoClAuNPsnGr) electrodes. The CV method was applied in order to assess the electrochemical response of the PIX/AuNPsnGr and PIXCoCl/AuNPsnGr. The CVs displayed in Figure 2a were recorded in a solution containing 5.0 × 10^−3^ mol L^−1^ K_3_[Fe(CN)_6_] (0.1 mol L^−1^ KCl) throughout a potential range of −0.6 to 1.0 V, at a scan rate of 0.1 V s^−1^, using AuNPsnGr, PIX/AuNPsnGr, and PIXCoCl/AuNPsnGr as working electrodes. When compared to the unmodified sensor, the modified 3D sensors exhibit a higher conductivity. According to these findings, the modifications with both PIX and PIXCoCl result in an improvement in the electrochemical response.

In order to conduct an analysis of the interfaces of the bare and modified electrodes using the EIS method, the frequency range for the analysis was between 10^5^ and 10^−1^ Hz. The experiments were carried out in a solution containing 5.0 × 10^−3^ mol L^−1^ of K_3_[Fe(CN)_6_] and 0.1 mol L^−1^ of KCl. Figure 2b displays the Nyquist plots obtained for AuNPsnGr (colored red), PIX/AuNPsnGr (colored dark), and PIXCoCl/AuNPsnGr (colored green). According to the Nyquist plot, AuNPsnGr exhibits a large, well-defined semicircle at low frequencies, which matches to a high electrical resistance (R_ct_ = 1899.0). This is due to the high electrical resistance of the material. Following the modification of AuNPsnGr with PIX and PIXCoCl, a semicircle with a reduced diameter (R_ct_ = 572.9) and a semicircle with a more noticeably reduced diameter (R_ct_ = 299.3), respectively, are recorded. This modification is successful. As both the diameter of the semicircle and the R_ct_ value are reduced, the electron transfer rate at PIX/AuNPsnGr and PIX/CoClAuNPsnGr surfaces is accelerated. The results of the EIS correspond adequately with the CV measurements.

The electrochemical behavior of AuNPsnGr, PIX/AuNPsnGr, and PIXCoCl/AuNPsnGr was further investigated utilizing the DPV method in pH 2.0 PBS containing 0.1 mol L^−1^ NaNO_3_ as a supporting electrolyte and 10^−9^ mol L^−1^ thiamine. In Figure 2c, the oxidation results of PIX/AuNPsnGr and PIXCoCl/AuNPsnGr for thiamine are greater than those of AuNPsnGr.

Calculating the electroactive surface area permitted the study of the electrocatalytic activity of the sensors, which was performed by applying the Randles–Sevick equation [20,21] to quasi-reversible redox processes controlled by diffusion. As demonstrated below, the peak current intensity on both the anodic and cathodic peaks is directly proportional to the square root of the scan rate:

$$Ip=\left(2.69\times 10^{5}\right)n^{3/2}AC_{0}D_{R}^{1/2\;}\nu^{1/2}$$

where *Ip* is the peak current (µA), *n* is the number of transferred electrons (*n* = 1, in this example), *A* is the electrode active surface area (cm^2^), *C*_0_ is the concentration of K_3_[Fe(CN)_6_] (mol cm^−3^), D_R_ is the diffusion coefficient (7.60 × 10^−6^ cm^2^ s^−1^), and *υ* is the scan rate (V s^−1^).

**Figure 3:**
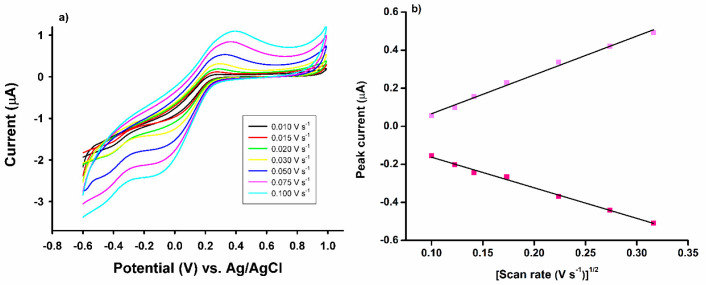
(**a**) CVs in a solution of 5.0 × 10^−3^ mol L^−1^ K_3_[Fe(CN)_6_] (0.1 mol L^−1^ KCl) at various scan rates ranging from 0.010 to 0.100 V s^−1^ using the PIX/AuNPsnGr sensor (working conditions: potential range from −0.6 to 1.0 V; step potential 0.025 V). (**b**) Dependence of the peak current on the square root of the scan rate for the PIX/AuNPsnGr sensor.

**Figure 4:**
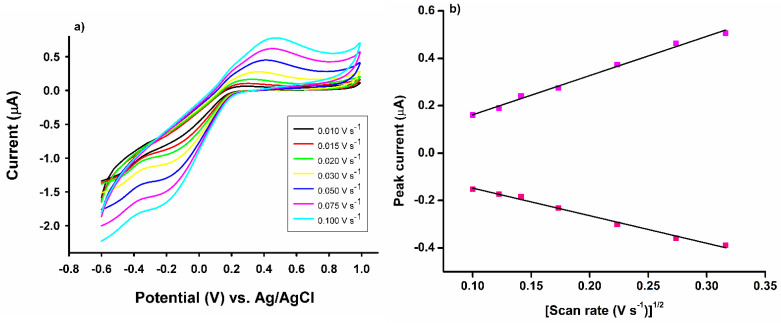
(**a**) CVs in a solution of 5.0 × 10^−3^ mol L^−1^ K_3_[Fe(CN)_6_] (0.1 mol L^−1^ KCl) at various scan rates ranging from 0.010 to 0.100 V s^−1^ using the PIXCoCl/AuNPsnGr sensor (working conditions: potential range from −0.6 to 1.0 V; step potential 0.025 V). (**b**) Dependence of the peak current on the square root of the scan rate for the PIXCoCl/AuNPsnGr sensor.

The fact that the anodic and cathodic peaks, Ip_a_ (purple dots) and Ip_c_ (pink dots), respectively, exhibit a linear dependence on the square root of the scan rate (Figure 3 and Figure 4) while the scan rate varies from 0.010 to 0.100 V s^−1^ indicates that the redox process is controlled by diffusion. Figure 3a and Figure 4a illustrate the pattern that emerges as the scan rate and current intensity both continue to increase, whereas Figure 3b and Figure 4b depict the linear dependences of the two peaks, *Ip_a_* vs. *υ*^1/2^ and *Ip_c_* vs. *υ*^1/2^, respectively. The sensor based on PIXCoCl/AuNPsnGr exhibits the highest active area (7.23 × 10^−4^ cm^2^) when compared to the other modified sensor, PIX/AuNPsnGr (5.58 × 10^−4^ cm^2^), and when compared to the sensor AuNPsnGr that has not been modified (4.53 × 10^−4^ cm^2^).

### 3.3. The Optimization of the pH Values and the Supporting Electrolyte

**Figure 5:**
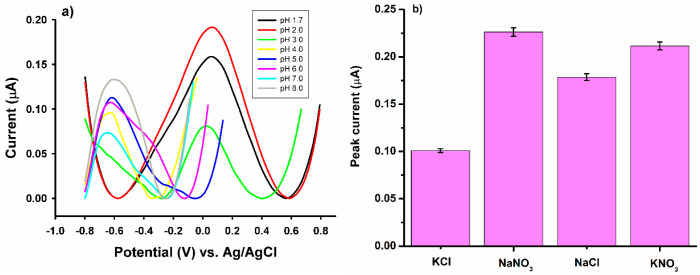
(**a**) DPVs of 10^−4^ mol L^−1^ thiamine in PIX/AuNPsnGr in PBS at pH values of 1.7, 2.0, 3.0, 4.0, 5.0, 6.0, 7.0, and 8.0; (**b**) the influence of the supporting electrolyte (0.1 mol L^−1^ NaCl, KCl, NaNO_3_, and KNO_3_) on the electrooxidation of 10^−4^ mol L^−1^ thiamine (pH 2.0 PBS).

**Figure 6:**
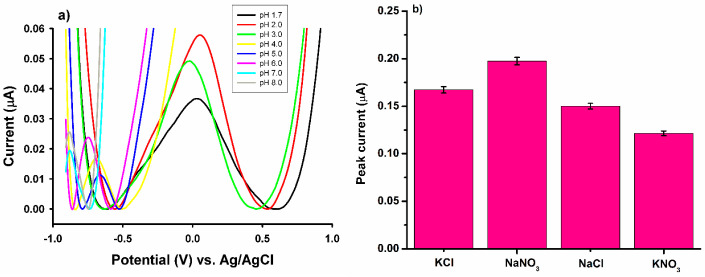
(**a**) DPVs of 10^−4^ mol L^−1^ thiamine in the PIXCoCl/AuNPsnGr in PBS at pH values of 1.7, 2.0, 3.0, 4.0, 5.0, 6.0, 7.0, and 8.0; (**b**) the influence of the supporting electrolyte (0.1 mol L^−1^ NaCl, KCl, NaNO_3_, and KNO_3_) on the electrooxidation of 10^−4^ mol L^−1^ thiamine (pH 2.0 PBS).

To achieve the best possible results, research was conducted to determine how pH and supporting electrolytes influenced the oxidation of thiamine. Analyses were performed on solutions of PBS with varying pH values, ranging from 1.7 to 8.0, and comprising 10^−4^ mol L^−1^ of thiamine as part of the study on the influence of pH. The maximum oxidation peak of thiamine is obtained in an acidic medium, as shown in Figure 5a and Figure 6a (pH 2.0). As a result of this, pH 2.0 PBS was utilized for all of the subsequent measurements.

To determine the effect of the supporting electrolyte on the electrooxidation of thiamine, different electrolyte solutions (0.1 mol L^−1^ of NaCl, KCl, NaNO_3_, and KNO_3_) were added to a solution of pH 2.0 PBS containing 10^−4^ mol L^−1^ of thiamine. When using 0.1 mol L^−1^ NaNO_3_, the peak current is the highest; hence, this is the best supporting electrolyte. This can be seen in Figure 5b and Figure 6b. As a result of this, all subsequent thiamine working solutions were prepared using 0.1 mol L^−1^ of NaNO_3_ as a supporting electrolyte.

### 3.4. Response Characteristics of the 3D Sensors in Differential Pulse Voltammetry Mode

**Figure 7:**
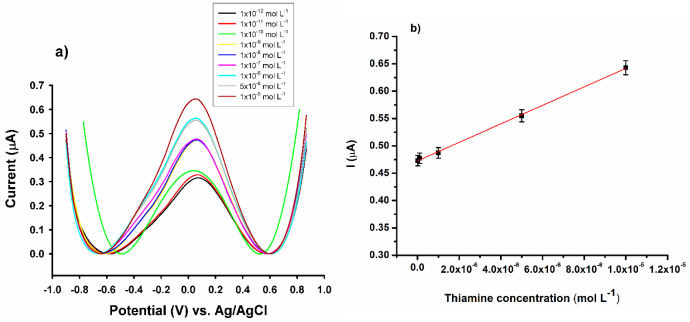
(**a**) Differential pulse voltammograms recorded with PIX/AuNPsnGr sensor in PBS pH 2.0 and 0.1 mol L^−1^ NaNO_3,_ containing different concentrations of thiamine from 1.0 × 10^−12^ mol L^−1^ to 1.0 × 10^−5^ mol L^−1^. (**b**) Calibration curve obtained with PIX/AuNPsnGr sensor from 1.0 × 10^−12^ mol L^−1^ to 1.0 × 10^−5^ mol L^−1^. Working conditions: step potential 0.025 V, amplitude: 0.1 V.

The response characteristics of the presented 3D electrochemical sensors were identified using differential pulse voltammetry (at the optimal pH level of 2.0), and they can be found in Table 1. Due to the optimal working conditions and electrocatalytic capacity of the porphyrins utilized in their design, the proposed 3D sensors are able to achieve a broad concentration range, high sensitivities, and low quantification and detection limits. Figure 7b shows the calibration graph for thiamine, along with the peaks that are produced during the calibration of the PIX/AuNPsnGr sensor. With a correlation coefficient of 0.9916, the linear concentration range is from 1.0 × 10^−12^ mol L^−1^ to 1.0 × 10^−5^ mol L^−1^. Both the limits of detection (LOD) and the limits of quantification (LOQ) are determined to be 3.0 × 10^−13^ mol L^−1^ and 1.0 × 10^−12^ mol L^−1^, respectively The following are the LOD and LOQ values: LOD = 3 s/m and LOQ = 10 s/m; where s is the standard deviation of the peak current (10 measurements) of the blank and m is the slope of the calibration curve. The PIX/AuNPsnGr sensor has a sensitivity of 1.49 × 10^8^ A mol L^−1^.

**Figure 8:**
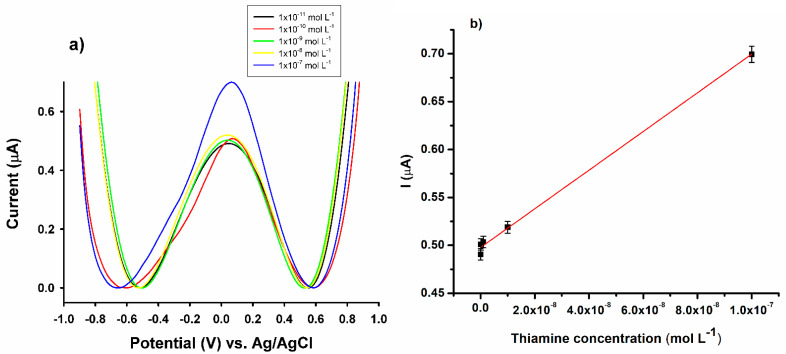
(**a**) Differential pulse voltammograms recorded with PIXCoCl/AuNPsnGr sensor in PBS pH 2.0 and 0.1 mol L^−1^ NaNO_3,_ containing different concentrations of thiamine from 1.0 × 10^−11^ mol L^−1^ to 1.0 × 10^−7^ mol L^−1^. (**b**) Calibration curve obtained with PIXCoCl/AuNPsnGr sensor from 1.0 × 10^−11^ mol L^−1^ to 1.0 × 10^−7^ mol L^−1^. Working conditions: step potential 0.025 V, amplitude: 0.1 V.

In relation to the PIXCoCl/AuNPsnGr sensor, Figure 8 depicts the peaks acquired (Figure 8a) following calibration measurements, as well as the calibration graph (Figure 8b). For calculating LOD and LOQ, the aforementioned formulas were utilized. Therefore, excellent findings are obtained: linear concentration range from 1.0 × 10^−11^ mol L^−1^ to 1.0 × 10^−7^ mol L^−1^, LOD and LOQ values determined to be 3.0 × 10^−12^ mol L^−1^ and 1.0 × 10^−11^ mol L^−1^, respectively, correlation coefficient of 0.9975, and sensitivity of 2.02 × 10^6^ A mol L^−1^.

### 3.5. Studies of the Interference That Occurs with the 3D Electrochemical Sensors

Vitamin B12, ascorbic acid, maltodextrin, fructose, glucose, FeSO_4_, CH_3_COONa, and NH_4_Cl were subjected to a series of tests in an effort to identify whether or not they interfered with the detection of thiamine. The molecules frequently identified with thiamine in blueberry syrup, multivitamin tablets, water, and urine samples were chosen as possible interfering substances. The tolerance limit was defined as the highest interference concentration that caused a change in current intensity in terms of relative error (5% acceptance level), bias (%), and signal change (%). All measurements were conducted with pH 2.0 thiamine solutions that were buffered with PBS at a concentration of 1.0 × 10^−9^ mol L^−1^ and 0.1 mol L^−1^ NaNO_3_. Experiments demonstrate that the employment of the PIX/AuNPsnGr sensor has no influence on the detection of thiamine, despite the addition of 10-fold CH_3_COONa, 25-fold NH_4_Cl, 50-fold FeSO_4_, fructose, and 100-fold glucose, maltodextrin, ascorbic acid, and vitamin B12 (Table 2). This demonstrates that the suggested sensor has a good selectivity for thiamine determination.

Experiments reveal that the addition of 25-fold NH_4_Cl, 50-fold FeSO_4_, 100-fold ascorbic acid, fructose, glucose, CH_3_COONa, maltodextrin, and vitamin B12 has no influence on the detection of thiamine using the PIXCoCl/AuNPsnGr sensor (Table 3). This demonstrates that the suggested sensor has a good selectivity for thiamine determination.

### 3.6. Reproducibility, Repeatability, and Stability

**Figure 9:**
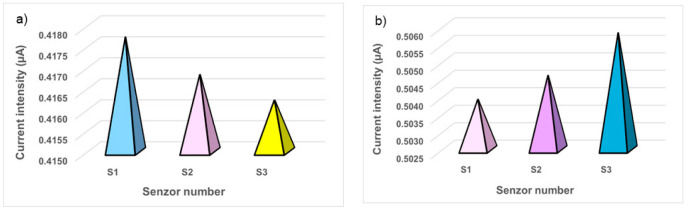
Reproducibility of the (**a**) PIX/AuNPsnGr and (**b**) PIXCoCl/AuNPsnGr.

The repeatability, reproducibility, and stability of the designed 3D sensors (PIX/AuNPsnGr and PIXCoCl/AuNPsnGr) were studied by DPV using a solution of thiamine (1.0 × 10^−9^ mol L^−1^) in PBS pH 2.0 and 0.1 mol L^−1^ NaNO_3_, under the ideal experimental conditions (Figure 9). Using three identically made new sensors of each type, the reproducibility was studied. It is determined that the relative standard deviation (RSD%) for the PIX/AuNPsnGr sensor is 0.15% (*n* = 3), whereas the PIXCoCl/AuNPsnGr sensor has an RSD% of 0.16% (*n* = 3).

**Figure 10:**
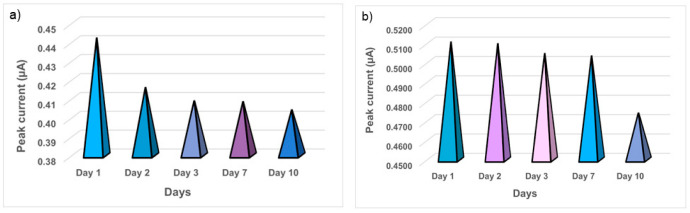
Stability of the (**a**) PIX/AuNPsnGr and (**b**) PIXCoCl/AuNPsnGr sensors for a period of 10 days.

Repeatability within a single day is determined to be 4.31% (*n* = 10) for the PIX/AuNPsnGr sensor and 3.21% (*n* = 10) for the PIXCoCl/AuNPsnGr sensor. Ten days were used to test the sensors’ stability (Figure 10). During the whole stability study, the modified electrodes were stored at 2–8 °C. After 10 days, for the PIX/AuNPsnGr sensor, the current intensity of thiamine (1.0 × 10^−9^ mol L^−1^) in PBS pH 2.0 and 0.1 mol L^−1^ NaNO_3_ decreases to 91.38% of the initial value from the first day of analysis; and for the PIXCoCl/AuNPsnGr sensor, the peak current also decreases to 92.82% of the initial value from the first day of analysis. The number of sensors used to determine reproducibility is *n* = 3. In contrast, *n* = 10 denotes the number of determinations in the case of repeatability and stability.

### 3.7. Determination of Thiamine in Food, Water and Biological Samples

Prior to their widespread use in the monitoring of food quality and control of food security, the suggested 3D sensors must be validated. Standard addition was used to test the 3D sensors’ capability to reliably assess thiamine in blueberry syrup, multivitamin tablets, water, and urine samples. This method was utilized to present the precision of 3D sensors. As shown in Table 4 and Table 5, the reproducibility of the thiamine analysis in blueberry syrup, multivitamin tablets, water, and urine samples is quite high.

After inserting the 3D sensors into the samples, the peak current was measured. The aforementioned calibration equation was used to calculate the thiamine concentrations. Recovery, RSD, and bias (%) results are displayed in Table 4 and Table 5.

The employment of the PIX/AuNPsnGr sensor results in the outcomes of excellent recovery values, as shown in Table 4. The recoveries for both the water and food samples are above 95%. In addition, the urine sample has a recovery value greater than 100%. RSD values for the samples range from 0.07 to 3.04. In the instance of the PIXCoCl/AuNPsnGr sensor, Table 5 reveals that the recoveries in water, food, and urine samples are greater than 95%, with RSD values ranging between 0.19 and 2.01. Consequently, based on the fact that Table 4 and Table 5 contain very good values, it can be stated that both sensors, PIX/AuNPsnGr and PIXCoCl/AuNPsnGr, demonstrate that they are able to provide better outcomes for sensitivity and selectivity of the assay for thiamine in food, water, and biological sample.

Table 6 shows that compared with other electrochemical methods proposed in the literature for the assay of thiamine, the 3D sensors present wider linear concentration ranges, as well as lower limits of detection.

## 4. Conclusions

The technological application of nanographenes will undoubtedly pave the way for the development of progressively more sensitive electrochemical sensors. Nanographenes are fascinating new instruments that, perhaps, have the potential to enhance food safety and quality control monitoring. In the perspective of such, the present study offers a mini-platform comprising two 3D electrochemical sensors that were developed, characterized, tested, and validated for the assessment of thiamine in blueberry syrup, multivitamin tablets, water, and a biological sample. Based on AuNPs and nGr paste, these 3D sensors were modified with PIX and PIXCoCl. Both sensors display extraordinarily high levels of stability, selectivity, sensitivity, and reproducibility in their respective studies. The suggested 3D sensors have the advantage of being able to be employed in the analysis of food, pharmaceutical, water, and biological samples in relation to the detection of the amount of thiamine contained by those samples.

## Figures and Tables

**Table 1:** Response characteristics of the 3D electrochemical sensors used for detection of thiamine.

Parameter	Three-Dimensional Electrochemical Sensors	
	PIX/AuNPsnGr	PIXCoCl/AuNPsnGr
Equation of calibration	Ip_a_ = 0.32 + 1.49 × 10^8^ × C_thiamine_, R^2^ = 0.9916	Ip_a_ = 0.50 + 2.02 × 10^6^ × C_thiamine_, R^2^ = 0.9975
Linear concentration range (mol L^−1^)	1.0 × 10^−12^–1 × 10^−5^	1.0 × 10^−11^–1.0 × 10^−7^
Sensitivity (A mol L^−1^)	1.49 × 10^8^	2.02 × 10^6^
Limit of detection (mol L^−1^)	3.0 × 10^−13^	3.0 × 10^−12^
Limit of quantification (mol L^−1^)	1.0 × 10^−12^	1.0 × 10^−11^
Repeatability of peak current (%, RSD)	4.31	3.21

**Table 2:** The influence of possible interfering species on the detection of 1.0 × 10^−9^ thiamine (*n* = 6) using the PIX/AuNPsnGr sensor.

Possible Interfering Species	Tolerance Limit	Signal Changed (%)	Relative Error (%)	Bias (%)
Ascorbic acid	100	0.37	−0.37	0.93
CH_3_COONa	10	−0.95	0.96	−1.70
FeSO_4_	50	−4.25	4.44	−4.88
Fructose	50	0.13	−0.13	0.81
Glucose	100	4.88	−4.65	6.42
Maltodextrin	100	−3.28	3.39	−6.87
NH_4_Cl	25	0.81	−0.80	3.61
Vitamin B12	100	−1.14	1.15	−5.16

**Table 3:** The influence of possible interfering species on the detection of 1.0 × 10^−9^ thiamine (*n* = 6) using the PIXCoCl/AuNPsnGr sensor.

Possible Interfering Species	Tolerance Limit	Signal Changed (%)	Relative Error (%)	Bias (%)
Ascorbic acid	100	1.00	−0.99	1.00
CH_3_COONa	100	−2.91	3.00	−0.84
FeSO_4_	100	0.84	−0.83	2.44
Fructose	100	2.56	−2.50	1.15
Glucose	25	−1.62	1.65	1.43
Maltodextrin	50	−2.32	2.38	−2.32
NH_4_Cl	100	−0.30	0.30	−2.56
Vitamin B12	100	−1.93	1.96	5.63

**Table 4:** Determination of thiamine in food and biological samples using the PIX/AuNPsnGr sensor.

Samples	Amount Added (mol L^−1^)	Amount Found (mol L^−1^)	Recovery (%)	RSD (%)	Bias (%)
Water 1	-	4.80 × 10^−10^	-	-	-
	1.0 × 10^−8^	1.04 × 10^−8^	100.03	1.03	−3.47
	1.0 × 10^−9^	9.90 × 10^−10^	99.02	0.07	0.99
Water 2	-	5.55 × 10^−10^	-	-	-
	1.0 × 10^−8^	1.01 × 10^−8^	99.97	0.61	−1.44
	1.0 × 10^−9^	1.01 × 10^−8^	100.84	0.01	−0.83
Water 3	-	5.44 × 10^−10^	-	-	-
	1.0 × 10^−8^	9.81 × 10^−9^	98.07	0.40	1.97
	1.0 × 10^−9^	9.94 × 10^−10^	99.39	0.16	0.61
Multivitamin tablet	-	1.02 × 10^−9^	-	-	-
	1.0 × 10^−8^	1.01 × 10^−8^	101.20	0.15	−1.19
	1.0 × 10^−9^	9.93 × 10^−10^	99.26	3.04	0.75
Blueberry syrup	-	1.38× 10^−9^	-	-	-
	1.0 × 10^−8^	9.58 × 10^−9^	95.79	1.49	4.39
	1.0 × 10^−9^	9.73 × 10^−9^	97.33	1.38	2.74
Urine	-	−2.01× 10^−9^	-	-	-
	1.0 × 10^−8^	1.02 × 10^−8^	99.96	2.18	−1.83
	1.0 × 10^−9^	1.02 × 10^−9^	99.98	0.36	−2.37

**Table 5:** Determination of thiamine in food and biological samples using the PIXCoCl/AuNPsnGr sensor.

Samples	Amount Added (mol L^−1^)	Amount Found (mol L^−1^)	Recovery (%)	RSD (%)	Bias (%)
Water 1	-	2.32 × 10^−8^	-	-	-
	1.0 × 10^−8^	1.02 × 10^−8^	101.85	2.01	−1.82
	1.0 × 10^−9^	9.80 × 10^−10^	97.99	0.99	2.05
Water 2	-	1.38 × 10^−8^	-	-	-
	1.0 × 10^−8^	1.02 × 10^−8^	100.18	0.69	−2.28
	1.0 × 10^−9^	1.01 × 10^−9^	99.95	1.51	−1.10
Water 3	-	2.36 × 10^−9^	-	-	-
	1.0 × 10^−8^	9.60 × 10^−9^	95.98	0.19	4.19
	1.0 × 10^−9^	9.96 × 10^−10^	99.62	0.45	0.38
Multivitamin tablet	-	1.02 × 10^−8^	-	-	-
	1.0 × 10^−8^	1.01 × 10^−8^	100.60	0.03	−0.59
	1.0 × 10^−9^	1.03 × 10^−9^	99.93	1.42	−3.00
Blueberry syrup	-	9.05 × 10^−9^	-	-	-
	1.0 × 10^−8^	1.01 × 10^−8^	99.98	0.19	−1.24
	1.0 × 10^−9^	1.03 × 10^−9^	99.93	0.98	−2.53
Urine	-	3.89 × 10^−9^	-	-	-
	1.0 × 10^−8^	1.03 × 10^−8^	100.97	1.24	−2.80
	1.0 × 10^−9^	9.75 × 10^−10^	97.47	1.25	2.60

**Table 6:** Comparison of the new developed method of analysis of thiamine (using 3D sensors) with other methods presented in the literature.

Method	Linear Concentration Range	Limit of Detection	Reference
SWV	15–110 µg mL^−1^	3.5 µg mL^−1^	[22]
DPAdSV	0.0025–0.80 g mL^−1^	1.1 ng mL^−1^	[23]
CV	10^−5^–10^−3^ mol L^−1^	5.34 × 10^−6^ mol L^−1^	[24]
CV	0.256–10 m mol L^−1^	6.9 × 10^−5^ mol L^−1^	[4]
CV	1.0 × 10^−12^–1 × 10^−5^ mol L^−1^	3.0 × 10^−13^ mol L^−1^	This work
CV	1.0 × 10^−11^–1.0 × 10^−7^ mol L^−1^	3.0 × 10^−12^ mol L^−1^	This work

## Data Availability

Not applicable.
